# Thinking out of the (Olympic) box: is it time to reconsider permanent hosting for sustainable Olympics? A conceptual review

**DOI:** 10.3389/fspor.2026.1778441

**Published:** 2026-05-01

**Authors:** Athanasios (Sakis) Pappous

**Affiliations:** Department of Quality of Life Studies, University of Bologna, Rimini, Italy

**Keywords:** institutional governance, international Olympic committee (IOC), legacies, Olympic games, paralympic games, sport mega events, sustainability, wicked problems

## Abstract

The modern Olympic Games confront a sustainability crisis that a half-century of incremental reforms has failed to resolve. This conceptual paper questions the entrenched “business-as-usual” logic of rotational Olympic hosting—a model established over a century ago under vastly different economic and environmental realities—and invites academic and policy communities to reconsider structural alternatives. Drawing on Rittel and Webber’s (1973) framework, we characterize Olympic sustainability as a “wicked problem” whose complexity, stakeholder pluralism, and structural contradictions resist incremental solutions. Empirical evidence reveals systematic patterns: every Olympic Games since 1968 has exceeded its original budget, sustainability performance has declined rather than improved over recent decades, and the rapid post-Games dissolution of organizing committees creates governance gaps that systematically undermine legacy delivery. We argue these persistent failures stem from a “construction imperative” inherent to rotation: cyclical mega-infrastructure development generates predictable economic dysfunction, environmental degradation, and “white elephant” venues regardless of host city competence or reform initiatives. This conceptual review explores permanent or semi-permanent hosting as a transformative structural alternative that could eliminate redundant construction, redirect resources toward global athlete development, and transform venues into year-round high-performance hubs accessible to all nations. By examining historical precedents—including Greece’s 1980 Karamanlis Plan for an extraterritorial Olympic territory—and engaging counter-arguments regarding equity and feasibility, we demonstrate that reconsidering the rotational model is not a radical departure from tradition. Instead, this analysis serves as a timely catalyst for the global sports community to “think out of the box” and evaluate alternative structural configurations that might better safeguard the Olympic ideal for future generations.

“If you have always done it that way, it is probably wrong.” Charles Kettering

## 1 Introduction: the systemic nature of Olympic challenges

The modern Olympic Games embody a profound paradox. Conceived by Baron Pierre de Coubertin as a vehicle for international understanding, peace, and athletic excellence, the Games have evolved into what economists describe as a “money-losing proposition” [(1), p. 201], what environmental scientists characterize as exhibiting “declining sustainability performance” [(2), p. 340], and what political scientists identify as “repeatedly lead[ing] to political disruptions and boycotts” [(3), p. 148]. The institution created to unite humanity has become inextricably linked with division, sovereign debt, and environmental degradation, threatening its long-term viability. This persistence of challenges across diverse contexts—different nations, political systems, economic conditions, and decades—suggests that the problems stem from structural features of the Olympic model itself rather than from inadequate oversight, raising fundamental questions about whether incremental reforms can ever be sufficient.

Empirical evidence reveals patterns suggesting these are not isolated failures but systemic pathologies embedded in the rotational hosting model. Every Olympic Games from 1968 to 2012 exceeded its original budget, with average cost overruns exceeding 150 percent—ranging from 156% to 179% depending on the dataset and time period analyzed (1, 4). Recent Games have reached absolute costs that challenge the economic rationality of hosting: Beijing 2008 spent $40 billion, Sochi 2014 reached $55 billion, and Tokyo 2020 reached $15.4 billion—with some estimates approaching $20 billion—despite pandemic-related reductions in spectator services (1, 5–7). The 16 Summer and Winter Games between 1992 and 2020 collectively cost tens of billions of dollars, yet sustainability assessments found declining performance over time rather than improvement (2). Khraiche and Alakshendra (8) document that “in the last 50 years, every Olympics has experienced a major cost overrun” (p. 845), establishing this as a systemic pattern rather than an occasional failure attributable to poor planning in specific host cities.

The pattern is remarkably consistent. It spans both pluralistic and centralized governance models, as well as wealthy and developing nations. This consistency demands an explanation beyond individual host city competence. Instead, it points to deep structural features of the rotational model that generate predictable failures regardless of the host's political or economic context.

These persistent failures are compounded by structural governance challenges. Organizing committees typically dissolve within months of the Games. In fact, 90% of staff depart within one month and 99% within four months. This elimination of institutional memory occurs precisely when long-term legacy planning becomes critical (9).

This pattern extends beyond the Olympics to other mega-sporting events. For the 2027 Pan-American Games, Barranquilla, Colombia, was initially appointed as host but subsequently withdrew due to financial constraints. In the selection process that followed, Lima, Peru—which had recently hosted the 2019 Pan-American Games—successfully defeated Asunción, Paraguay, a city that had never hosted the event. This recent case demonstrates the implicit preference of selection committees for returning to proven hosts with existing infrastructure, driven by their capacity to deliver successful events at lower cost and risk compared to first-time hosts requiring extensive new construction. Peru's strategic advantage lay in its ability to reuse many venues from the 2019 Games, significantly reducing capital expenditure and environmental impact. This pragmatic turn toward venue reuse and proven hosts in the Pan-American context mirrors broader trends in Olympic hosting that we examine throughout this paper.

The consistency of these failures across diverse contexts raises fundamental questions about causation. Zimbalist, Solberg, and Storm (7) pose the existential question that frames this paper: might the Olympic sustainability crisis stem from a structural “principal-agent problem” inherent to the rotational model itself? In this framework, the International Olympic Committee (IOC) functions as the principal, capturing the majority of Olympic revenues through broadcasting and sponsorship rights while transferring financial risks to host cities (the agents). This asymmetric incentive structure creates a fundamental misalignment: the IOC benefits from increasingly spectacular Games regardless of cost, while host cities bear the financial consequences of overspending with uncertain returns. The competitive bidding process exacerbates this dynamic, as cities that present conservative, realistic cost estimates are systematically outbid by competitors promising grander visions. This principal-agent misalignment, combined with the rotational model's requirement for cyclical construction in varying geopolitical contexts, may constitute the structural foundation of Olympic sustainability challenges—suggesting that incremental reforms addressing symptoms may prove insufficient without confronting the underlying architecture of the model itself.

This conceptual paper explores the argument that the Olympic sustainability crisis requires transformative structural alternatives rather than incremental reforms. While questioning the rotational model may appear to be a radical departure from established Olympic tradition today, a historical retrospective reveals that geographic rotation was not always considered the only way to host the Games. In fact, it was fiercely debated at the movement's inception and has been repeatedly challenged during past eras of crisis. At the movement's birth in 1896, King George of Greece proposed permanent hosting in Athens. His vision was not merely nostalgic but strategic; he argued that permanent hosting in Greece would prevent the Olympics from becoming instruments of national propaganda, as Greece's relatively weak geopolitical position would preclude using the Games for great power competition. However, Baron Pierre de Coubertin firmly opposed this idea in favor of an “ambulatory” model designed to spread the Olympic message globally and establish international legitimacy, setting the rotational precedent that continues today (10).

This structural debate reemerged during a second historical moment in the 1970s and 1980s, a period of profound geopolitical and financial crises marked by the Munich terrorism, Montreal's massive debts, and Cold War boycotts. In response, Greek Prime Minister Constantine Karamanlis formally proposed a permanent Olympic site in 1976. The plan's most innovative feature was its proposal for extraterritorial, neutral status—analogous to the Vatican or United Nations headquarters—which would insulate the Games from domestic politics and the great power competition of the quadrennial bidding process (11). The proposal gained international support, including from American Senator Bill Bradley, who argued that competing nations could collectively underwrite permanent facilities rather than individual hosts bearing full costs. While the IOC ultimately rejected the plan in 1981, this decision was driven largely by a desire to preserve the geopolitical leverage and marketing revenue inherent in competitive bidding. These historical precedents demonstrate that evaluating permanent hosting is not a novel conceptual leap. Instead, it is a necessary continuation of a structural debate that surfaces whenever the movement faces existential pressures.

## 2 Theoretical framework: Olympic sustainability as a wicked problem

To understand why fifty years of Olympic reforms have failed to resolve sustainability challenges, we must first recognize the nature of the problem itself through the lens of “wicked problems” theory. Rittel and Webber (12) introduced this concept to describe challenges that resist conventional problem-solving approaches due to their inherent complexity, interconnectedness, and resistance to definitive solutions. Unlike “tame” problems that can be solved through technical optimization, wicked problems involve fundamental disagreements about problem definition, conflicting stakeholder values, and deep structural contradictions that cannot be resolved through better management alone. Byers, Hayday, and Pappous (13) have specifically applied this framework to mega sports event legacy, arguing that legacy delivery exhibits characteristics of wicked problems due to “the contested and complex nature of sport event legacy” and the “multiple characteristics of what constitutes mega events” (p. 173). Their conceptualization challenges the “often utopian assumptions which position legacy as a positive way to generate additional (economic, social, and political) outcomes” [(13), p. 173] and instead encourages recognition of legacy as “problematic rather than merely a solution,” opening space for considering transformative alternatives rather than assuming that incremental improvements within the existing model will eventually succeed.

**Figure 1:**
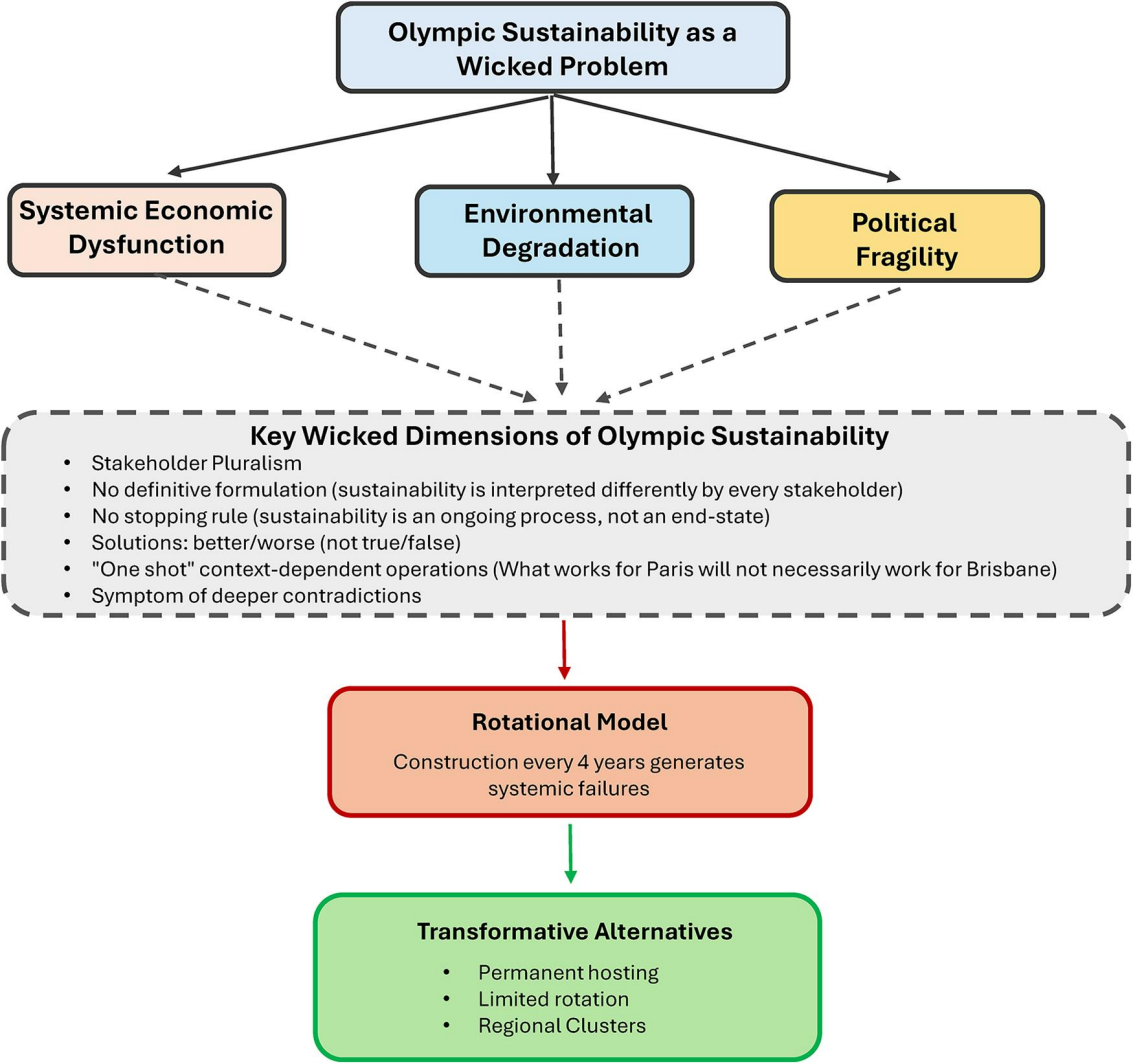
The Olympic sustainability as wicked problem and alternative hosting solutions..

The Olympic sustainability crisis exhibits some of the defining characteristics of wicked problems identified by Rittel and Webber (12) in ways that illuminate why the rotational model itself may be the source of systemic dysfunction (Figure 1).

Foremost among these is stakeholder pluralism, which ensures there is no definitive formulation of the problem and no neutral arbiter to determine which perspective should prevail. This dynamic is vividly illustrated by the ongoing (at the time of this writing) Milano-Cortina 2026 Games. While the IOC frames its high venue reuse rate as a triumph of sustainable delivery, coordinated coalitions of elite athletes and climate advocacy groups define the sustainability problem through structural climatic impacts, actively protesting the event's reliance on artificial snowmaking and fossil fuel sponsorships. For example, 88 Olympians published an open letter during the Milano-Cortina Games demanding an end to fossil fuel partnerships, highlighting the existential contradiction of celebrating human achievement while funding the event through industries that threaten the fundamental climatic conditions necessary for winter sports (14). Simultaneously, the geopolitical dimension of this wicked problem is exposed by the multi-nation boycott of the 2026 Milano-Cortina Paralympics opening ceremony over the inclusion of Russian and Belarusian athletes. This illustrates how rotating the Games inevitably entangles them in contemporary global conflicts.

Because solutions cannot be judged objectively as “true” or “false”—only as “better” or “worse” for different constituencies—any reform inevitably creates winners and losers. Furthermore, wicked problems have no stopping rule. The Olympic movement continuously implements reforms, yet each cycle reveals new challenges or unintended consequences, which creates “reform fatigue” and ensures the sustainability problem is never truly solved.

Crucially, each Olympic Games is essentially an irreversible “one-shot operation” [(12), p. 163]. Host cities cannot experiment with different approaches through trial-and-error without bearing enormous financial and social costs. By the time a Games' full impacts become apparent, the next host has already been selected based on similarly optimistic projections. Because every host context is uniquely constrained by distinct geopolitical and economic conditions, purported “best practices”—such as the privately financed 1984 Los Angeles Games—cannot be easily transferred to other cities. Ultimately, these surface-level failures are symptomatic of deeper structural contradictions between Olympic universalism and national prestige, or between sustainability rhetoric and the imperative for spectacular growth.

Byers et al. (13) emphasize that recognizing mega sports events as wicked problems requires examining “deep social structures which underpin different stakeholders” interpretations and interactions, which produce or limit legacy delivery” (p. 171). These deep structures—such as the competitive bidding process, asymmetric IOC-host incentives, and the political economy of mega-events—are embedded in the fundamental architecture of the rotational model. Because wicked problems resist incremental optimization, Rittel and Webber (12) suggest they may require not better management of existing systems, but transformation of the systems themselves. If the “construction imperative” embedded in rotation is the causal mechanism generating these intertwined crises, the question becomes not “How can we make the rotational model more sustainable?” but rather “What hosting arrangements might be compatible with genuine sustainability?” This reframing opens space for considering transformative alternatives, including the permanent hosting model that forms the focus of our subsequent analysis.

## 3 Structural limitations of the rotational hosting model

The theoretical framework suggests that Olympic sustainability challenges are systemic, a premise supported by five decades of empirical evidence revealing persistent dysfunction across economic, environmental, and political domains. Economically, the rotational model exhibits a systematic “winner's curse” driven by principal-agent misalignments. The IOC captures the majority of revenues while host cities bear the financial risks of competitive bidding, generating a structural incentive for bidders to systematically underestimate costs to win approval (7). Consequently, every Olympic Games from 1968 to 2012 exceeded its budget, with recent mega-events like Sochi 2014 and Tokyo reaching unsustainable absolute costs regardless of the host nation's economic or political context (1, 8).

Environmentally, this dysfunction is driven by the rotational model's “construction imperative.” The requirement to build or significantly upgrade venues every four years generates massive, redundant construction impacts and subsequent “white elephants”—facilities with limited post-Games utility that burden local economies (15). Despite increasingly sophisticated environmental management systems, overall sustainability performance has demonstrably declined over time (2). This crisis is particularly acute for the Winter Games; the rotational mandate forces the selection of increasingly marginal, climatically unsuitable locations that must rely on resource-intensive artificial snowmaking, paradoxically contributing to the very climate change that threatens winter sports (16).

Beyond economic and environmental deficits, the rotational model frequently undermines social sustainability by exacerbating inequalities within host cities. The inflexible deadlines and massive spatial requirements of Olympic preparation regularly necessitate the displacement of marginalized communities. These pressures also lead to rapid gentrification and the reallocation of public funds away from essential social services. This dynamic—observed across vastly different geopolitical contexts, from Beijing to Rio de Janeiro (17, 18)—disproportionately burdens vulnerable populations. Consequently, the rotational requirement often transforms Olympic hosting into a mechanism of spatial and social disruption, fundamentally contradicting the movement's stated commitment to human development.

Compounding these economic, environmental, and social costs is the systematic political fragility generated by the rotational model of hosting. By exposing each iteration of the Games to the specific geopolitical vulnerabilities and domestic controversies of its host nation, the model inherently subjects the Olympic movement to recurring cycles of boycotts, security escalations, and human rights disputes (3). This is not merely a historical Cold War artifact. The diplomatic boycotts and travel disruptions surrounding the recent 2026 Milano-Cortina Paralympics demonstrate this ongoing vulnerability. Driven by conflicts in Ukraine and the Middle East, these disruptions show how the rotational model continuously forces the global sports stage to navigate acute regional tensions. Ambitious incremental reforms like Olympic Agenda 2020 + 5 encourage venue reuse and shift away from open competitive bidding. Despite these efforts, the underlying structural requirement for cyclical mega-event mobilization ensures these multi-dimensional challenges remain unresolved. The persistent failure of these reform initiatives to arrest the decline in sustainability performance indicates that the root cause lies in the fundamental architecture of the rotational model itself.

## 4 Permanent hosting as a potential alternative

### 4.1 The logic of permanent hosting: overcoming the construction imperative

Having established that the rotational model's construction imperative generates systemic failures across economic, environmental, and political dimensions, and that incremental reforms have proven insufficient to arrest declining sustainability performance, we now examine permanent hosting as one potential transformative alternative that could address the root cause rather than symptoms of Olympic dysfunction. This section articulates how permanent hosting would eliminate the construction imperative, explores the economic and environmental logic of venue optimization rather than redundant construction, and examines how a permanent site could restore deeper Olympic values while enhancing rather than undermining global equity.

The fundamental advantage of permanent hosting lies in eliminating the construction imperative that drives the systemic challenges documented in Section 3 of this conceptual paper. Rather than building or significantly upgrading Olympic-scale infrastructure every four years in new locations—each time incurring massive construction costs, generating carbon emissions, creating geopolitical vulnerabilities, and producing facilities with uncertain post-Games utility—permanent hosting would concentrate infrastructure in one or two optimized locations that could be continuously refined based on lessons learned from regular use. This shift from redundant construction to continuous optimization addresses the causal mechanism identified as generating Olympic sustainability challenges: if rotation requires construction which generates costs, emissions, and “white elephants,” then eliminating rotation eliminates the construction imperative and its downstream consequences.

The economic logic of permanent hosting rests on transforming Olympic infrastructure from a one-time expense with limited post-Games value into a long-term asset with continuous utility. Under the rotational model, each host city builds Olympic facilities that are used intensively for 17 days and then must find post-Games purposes—often unsuccessfully. Under permanent hosting, facilities would be designed from the outset for year-round use, hosting not only the Olympics every four years but also World Championships, training camps, sports tourism, and educational programs in intervening years.

Tang et al. (15) demonstrate that successful transformation of Olympic venues into “positive legacies” requires careful planning for post-Games use, integration into existing urban infrastructure, and ongoing institutional support—conditions difficult to achieve when venues are built under the time pressure of Olympic deadlines in cities without established demand for Olympic-scale facilities. A permanent site could be designed from the beginning for optimal year-round functionality rather than being retrofitted for post-Games use.

Beyond direct venue efficiency, permanent hosting could generate substantial savings through the elimination of recurring costs. Each Olympic Games requires not only venue construction but also transportation infrastructure upgrades, security systems, telecommunications networks, media facilities, and administrative structures. Under rotation, these systems must be built or upgraded in each new host city. Under permanent hosting, these investments would be made once and then maintained and upgraded incrementally, dramatically reducing total costs over time.

The capital savings from permanent hosting could be reinvested globally to enhance Olympic equity rather than being concentrated in host cities. Currently, hosting the Olympics provides substantial benefits to the host city (infrastructure, global attention, tourism) while most nations receive only the intangible benefits of participation. If the billions saved through permanent hosting were redirected into athlete development programs, sports facility construction in developing nations, and grassroots sports participation worldwide, permanent hosting could actually enhance global equity compared to the current system that concentrates benefits in wealthy nations capable of hosting.

Beyond these immediate economic and environmental advantages, permanent hosting would enable the accumulation and intergenerational transfer of specialized organizational knowledge and workforce expertise. Under the rotational model, each host must build organizational capacity from scratch, recruiting and training thousands of staff and volunteers who acquire valuable expertise in Olympic operations, venue management, security protocols, and event coordination. However, as recent qualitative analyses demonstrate, this ephemeral governance structure—where Organizing Committees (OCOGs) operate independently and dissolve almost immediately after the closing ceremony—structurally prioritizes short-term operational delivery over long-term urban integration (9). Consequently, this organizational knowledge quickly dissipates after the Games conclude, lost to the broader Olympic movement as personnel return to other careers. Permanent hosting would transform this pattern of cyclical knowledge loss into a model of cumulative organizational learning, where both paid staff and volunteer corps develop deep expertise refined across successive Games. This institutional memory would encompass not only technical operations but also the tacit knowledge gained through experience—the subtle understanding of how to anticipate challenges, coordinate complex systems, and maintain excellence under pressure. The organizational knowledge would be incrementally built and systematically passed from one generation to the next, creating a professional Olympic workforce whose expertise serves the global movement rather than being repeatedly reconstructed and abandoned. This continuity would enhance operational excellence while reducing the risks inherent in entrusting each Games to newly formed organizational structures with limited Olympic experience.

The environmental logic of permanent hosting is equally compelling, as it eliminates the redundant construction that Müller et al. (2) identify as the primary driver of declining Olympic environmental performance despite increasingly sophisticated management systems. Construction generates massive carbon emissions from materials production, transportation, and site preparation; consumes resources including water, energy, and raw materials; disrupts ecosystems and displaces communities; and creates waste both during construction and when facilities are eventually demolished or abandoned. The rotational model multiplies these impacts by requiring construction every four years in new locations, overwhelming any marginal improvements from “green” building techniques or better environmental management. Permanent hosting would incur construction impacts once (for initial build-out) rather than repeatedly, then focus environmental efforts on optimizing operations rather than mitigating construction—renewable energy systems, water conservation, waste reduction, sustainable transportation, and ecosystem restoration could be implemented and refined over time rather than being compromised by the urgency and constraints of preparing for a one-time event.

For Winter Olympics, the environmental and climatic logic is particularly strong given the challenges documented by Dean (16) regarding declining snow reliability and increasing artificial snow dependence. Permanent hosting in climatically optimal locations—regions with high altitude, reliable natural snowfall, and favorable latitude—would eliminate the need for massive artificial snow production that characterizes marginal Winter Olympic sites, reducing water and energy consumption while ensuring reliable conditions for athletes and spectators. The Alpine region, for example, includes peaks above 4,000 meters that will remain snow-reliable even under continued warming scenarios, while many recent and potential Winter Olympic hosts are already experiencing snow reliability problems that will only worsen. Concentrating Winter Olympics where natural conditions are optimal rather than rotating through increasingly marginal sites addresses both the environmental costs of artificial snow and the competitive integrity concerns of holding winter sports in locations without reliable winter conditions.

Permanent hosting also addresses concerns about the IOC's revenue redistribution model and global equity in ways that may be counterintuitive but are crucial to understanding the proposal's implications. The IOC currently functions as a global redistributor, returning approximately 90 percent of its revenue to National Olympic Committees (NOCs) and International Federations (IFs) worldwide, and this redistribution model is fundamental to the IOC's role in the global Olympic movement (19). Under the rotational model, billions of dollars are lost to lost to unsustainable capital expenditure, the repeated construction of venues which frequently fall into underutilization. A permanent venue model would eliminate this waste and redirect those billions toward the IOC's global redistribution system—rather than each host city spending $10–50 billion on infrastructure that serves limited post-Games purposes, a permanent site would require significant initial investment but dramatically lower recurring costs, with the savings from eliminating redundant construction allocated to athlete development programs, training facilities, and competitive opportunities in under-resourced nations. This represents a shift from a model where individual wealthy cities bear costs for temporary prestige toward a model of collective investment in shared, optimized infrastructure that serves the global Olympic community, potentially doubling or tripling the support currently provided to NOCs in developing nations.

Furthermore, a permanent Olympic site could function as a global high-performance hub accessible to athletes from all nations, particularly those from countries lacking advanced training facilities, transforming the site from a national asset into a shared global utility. Rather than sitting dormant between Games, the permanent site could operate year-round as an international training center. Here, athletes from developing nations could access world-class facilities through Olympic scholarships and development programs. This addresses a profound inequity in the current model, where advanced training facilities are concentrated in wealthy nations while athletes from developing nations lack comparable resources. This global high-performance hub model has precedents in other international sporting contexts (the Olympic Training Center in Colorado Springs provides year-round facilities for U.S. athletes but also hosts international training camps and competitions), and a permanent Olympic site could expand this model globally, providing facilities accessible to athletes from all nations rather than just the host nation. This would demonstrate that permanent hosting serves global rather than regional interests, with the site functioning as a shared resource for the entire Olympic movement rather than as a monopoly.

The case for permanent hosting extends beyond economic efficiency and environmental sustainability to encompass deeper questions about the Olympic movement's cultural and spiritual significance in an era of mounting challenges to its legitimacy. The Olympics confront not only fiscal and ecological crises but also threats to their unique identity: the proliferation of doping scandals undermines fair competition; the emergence of alternative events like the “Enhanced Games” (which explicitly permit performance enhancement) challenges traditional Olympic values and generates controversy that threatens Olympic unity. In this context of multiple challenges, the Olympics must reinforce their claim to uniqueness—their status as something fundamentally different from, and more significant than, other sporting competitions. This requires not merely updated policies but a renewal of what Coubertin termed religio athletae—the “religion of athletics” that elevates the Olympics from mere competition to a secular ritual of global significance. A permanent Olympic site could serve this function by providing a “sacred space” (20), a physical embodiment of Olympic ideals that athletes and spectators experience as qualitatively different from ordinary sporting venues. This is not merely symbolic; it addresses a practical imperative. Athletes, as the Olympics' most crucial stakeholders, would become ambassadors not only of traditional Olympism but of evolving values continuously reinforced through their connection to a returning sacred place. The permanent site would function as a “temple” of athletics where new Olympic values are forged and transmitted across generations, providing the cultural continuity necessary to navigate contemporary challenges while maintaining the movement's distinctive identity. In this sense, permanent hosting addresses sustainability challenges that transcend economics and environment, touching the existential question of what makes the Olympics meaningful in the 21st century.

Indeed, beyond economic and environmental advantages, permanent hosting could address deeper questions of Olympic meaning, purpose, and values that have been eroded by the commercialization and politicization associated with the rotational model. Under the current system, Olympic stadiums function primarily as transient “media objects” designed for a two-week television broadcast before being abandoned or repurposed, lacking deeper cultural resonance (21). Permanent hosting would reverse this dynamic, allowing venues to accumulate the memories, traditions, and tangible history necessary to cultivate a sense of the sacred (20). Rather than competing in an anonymous stadium, athletes would perform in venues laden with the achievements of previous generations. Venues conceived as culturally significant spaces are more likely to be maintained, used, and valued by communities long after individual events conclude, serving practical sustainability purposes alongside their symbolic value. To bridge the gap between these theoretical benefits and practical application, it is necessary to explore how such permanent, culturally resonant sites might be operationalized in specific geographic contexts.

### 4.2 Illustrative models: Athens and the alpine cluster

We position Athens, the capital of Greece, and an Alpine cluster as illustrative examples for the Summer and Winter Games, respectively. These serve as thought experiments that operationalize the concept of reduced construction cycles, rather than as prescriptive solutions. Our purpose is to catalyze rigorous academic and institutional debate about a spectrum of transformative options that might address the sustainability challenges identified in previous sections. In doing so, we aim to reinvigorate a discourse that emerged simultaneously with the modern revival of the Games in 1896 and resurfaced during the crises of the 1970s and 1980s. Despite this rich historical precedent, critically questioning the rotational model has paradoxically become somewhat of an institutional taboo in contemporary Olympic management research. It is essential to emphasize that permanent hosting represents one option among several that could potentially reduce what we have termed the “construction imperative”—the requirement for extensive new building every four years. The specific locations and models discussed below are presented as heuristic devices to demonstrate practical feasibility and stimulate discussion, not to advocate for particular solutions or to suggest that any single approach represents the definitive answer to Olympic and Paralympic sustainability challenges.

#### 4.2.1 Athens as an illustrative model for summer olympics

Athens presents one potential option for exploring reduced-rotation or permanent Summer Olympic hosting, primarily due to its historical connection to the Games, existing infrastructure, and symbolic significance.

Importantly, permanent hosting in Athens is not a purely theoretical proposition—it represents a continuation of serious institutional proposals that have twice gained significant international consideration. As discussed earlier, King George of Greece proposed permanent hosting in Athens at the movement's inception in 1896, arguing it would prevent the Games from becoming instruments of national propaganda (10). This structural debate reemerged during the 1970s–1980s crisis period, when Prime Minister Constantine Karamanlis formally proposed a permanent Olympic site with extraterritorial neutral status in 1976, gaining support from international figures including U.S. Senator Bill Bradley before the IOC's rejection in 1981 (11). These precedents demonstrate that Athens has been evaluated not merely as a symbolic location but as a viable institutional alternative during previous eras of Olympic crisis. The governance mechanisms proposed in these historical plans—particularly the Karamanlis Plan's extraterritorial framework, which we examine in detail in Section 5.4—provide established models that could inform contemporary implementation, distinguishing this example from purely hypothetical alternatives.

The city's historical continuity—having hosted the ancient Olympic Games from 776 BCE for over a millennium, the inaugural modern Olympics in 1896, and the return Games in 2004—creates a narrative connection between the modern Games and their ancient origins that may resonate with Olympic values of tradition and continuity.

From a practical standpoint, the extensive infrastructure developed for the 2004 Games already exists and could potentially be repurposed for permanent or semi-permanent hosting rather than remaining underutilized. The Olympic Stadium, Velodrome, Aquatic Center, and other facilities were constructed to high standards and could, with appropriate maintenance and periodic upgrades, serve ongoing Olympic purposes (22). Athens' Mediterranean climate offers generally favorable conditions for summer athletics, while the city's accessibility from Europe, Asia, and Africa through established international airport and port facilities provides transportation infrastructure that could support a global mega-event. Greece's status as a medium-sized nation within the European Union may provide political stability without the great power dynamics that could complicate hosting in larger nations, though this remains a matter for political analysis rather than definitive assertion.

Beyond practical considerations, an Athens-based model could integrate the sacred site of ancient Olympia to reinforce the cultural and spiritual continuity that permanent hosting enables. While Athens possesses the infrastructure necessary for athletic competition, Olympia—the site where the ancient Games were held for over a millennium—offers the symbolic resonance that distinguishes the Olympics from ordinary sporting competitions. A hybrid approach could host primary athletic competitions in Athens while reserving key ceremonial elements for Olympia. For instance, athletes could take the Olympic Oath in the ancient stadium at Olympia, where the Olympic Flame remains throughout the Games, while the main competitions occur in Athens modern facilities. This integration would operationalize the concept of sacred space (20) by anchoring the Games in a physical location that has accumulated centuries of cultural meaning, rather than treating venues as transient media objects (21). The Karamanlis Plan historical proposal for a site near ancient Olympia (11) provides precedent for this integration, demonstrating that combining Athens practical infrastructure with Olympia sacred significance has been considered in previous institutional proposals.

However, we emphasize that Athens is presented here as one illustrative example among several possibilities and should not be interpreted as excluding other viable options or as suggesting that this represents the only or optimal solution.

#### 4.2.2 The alpine cluster as an illustrative model for winter Olympics

For Winter Olympics, the particular constraints imposed by climate change and the specific requirements of winter sports suggest that a different spatial approach may warrant consideration. Rather than proposing a single permanent city, a transnational Alpine cluster—spanning the cross-border mountain regions of Switzerland, Austria, Italy, France, Germany, and potentially Slovenia—serves as an illustrative model that could address the unique challenges of winter sports hosting while potentially maintaining some benefits of geographic concentration.

Winter Olympic sports require a complex combination of high-altitude mountain venues for snow-based events (alpine skiing, snowboarding, cross-country skiing) and urban infrastructure for ice sports, ceremonies, and accommodations. The Alpine region encompasses some of Europe's highest peaks, including Mont Blanc (4,808 meters), the Matterhorn (4,478 meters), and numerous other peaks above 4,000 meters, providing substantial vertical drops that could meet Olympic requirements for downhill skiing and other mountain events. Critically, research suggests that high-altitude Alpine locations may remain among the few European sites likely to maintain natural snow reliability even under continued warming scenarios, making them potentially more climatically suitable for Winter Olympic hosting than many lower-altitude locations currently under consideration (16).

The broader Alpine region already possesses established winter sports infrastructure developed over decades, including facilities from previous Winter Olympics: Chamonix 1924 (France), St. Moritz 1928 and 1948 (Switzerland), Cortina d'Ampezzo 1956 (Italy), Innsbruck 1964 and 1976 (Austria), the recent Milano-Cortina 2026 (Italy), and the forthcoming French Alps 2030 (France). Additionally, the region includes numerous world-class ski resorts and winter sports venues across all six countries that regularly host World Cup competitions and other international events. The proximity to major urban centers—including Geneva, Zürich, Innsbruck, Turin, Milan, Munich, and Lyon—provides substantial accommodation capacity and transportation hubs, while the region benefits from well-developed rail infrastructure connecting cities and mountain venues.

Encompassing the full Alpine arc offers several potential advantages. First, it provides greater geographic flexibility to select optimal venues based on natural conditions. Second, it distributes hosting responsibilities and benefits across multiple nations, reducing concerns about monopolization by any single country. Finally, it enhances the region's capacity to absorb the massive infrastructure requirements of the Winter Games. However, such a multi-national cluster would also present significant governance challenges, requiring unprecedented levels of cross-border coordination and cooperation that may prove difficult to achieve in practice.

It should be emphasized that the Alpine cluster, similarly to the Athens one, is presented as one illustrative model for addressing Winter Olympic sustainability challenges, not as a definitive prescription. Other climatically suitable regions—including parts of North America, Asia, or other mountain ranges—could potentially serve similar functions, and the specific geographic boundaries and governance structures of any such cluster would require extensive negotiation and analysis beyond the scope of this conceptual paper.

This section has articulated permanent hosting as a potential alternative that might address multiple dimensions of Olympic sustainability challenges while simultaneously reinforcing the construction of uniqueness, authenticity, and sacred significance that distinguishes the Olympics from ordinary sporting competitions. However, any serious consideration of permanent hosting must confront legitimate counter-arguments and practical challenges.

## 5 Challenges and counter-arguments

Any proposal for permanent Olympic hosting must address potential counter-arguments regarding Eurocentrism, loss of global character, feasibility challenges, and risks of long-term host fatigue. These critiques have merit and require substantive engagement rather than dismissal, as they raise fundamental questions about whether permanent hosting would enhance or undermine Olympic values, whether it is practically achievable, and what unintended consequences it might generate. This section engages these counter-arguments directly, examining the tension between concentration and universalism, assessing practical feasibility, and considering risks that permanent hosting might entail.

### 5.1 The eurocentrism critique and global equity

The most significant counter-argument is that permanent hosting in Athens and an Alpine cluster would concentrate the Olympics in Europe, potentially undermining Olympic universalism and excluding other regions from hosting opportunities, raising questions of equity and representation that go to the heart of Olympic values. This critique has particular force given the Olympics' stated commitment to global inclusivity and given historical patterns where global institutions have been dominated by Western powers to the exclusion of the Global South. If the Olympics are permanently located in Europe, does this not confirm that they are a European rather than global institution, and does this not exclude the vast majority of the world's population from meaningful participation in Olympic hosting and governance?.

However, this counter-argument must be evaluated against the reality that the current rotational model already exhibits profound inequities that permanent hosting could actually address rather than exacerbate. The rotational model in practice is already exclusive to wealthy nations capable of bearing Olympic costs—of the 30 Summer Olympics held since 1896, 23 have been in Europe or North America, with only 7 in Asia, Australia, and South America combined, and zero in Africa despite African nations comprising over 50 of the 200+ Olympic nations. Furthermore, the financial and infrastructural burden has escalated so dramatically that even within the Global North, the traditional competitive bidding process has experienced a severe contraction. For instance, Los Angeles will host the 2028 Olympics for the third time (after 1932 and 1984), winning the bid technically without an opponent; following a mass exodus of candidate cities, the IOC was forced in 2017 to broker an unprecedented joint agreement awarding the 2024 Games to Paris and the 2028 Games to Los Angeles, the only two cities remaining (23). By demanding massive, cyclical infrastructure investments, the rotational model imposes structural financial barriers that effectively price developing nations out of realistic hosting prospects. This dynamic creates a system where Olympic hosting functions as a consolidated capacity of the Global North rather than a genuinely universal opportunity. Consequently, the symbolic equity of geographic rotation among affluent nations fails to deliver substantive equity for the Global South. Instead, developing nations face compounded disadvantages: they are systematically marginalized from hosting opportunities, while the billions of dollars absorbed by redundant host-city infrastructure—often resulting in underutilized “white elephants”—represent a profound opportunity cost that diverts capital away from global athlete development.

Transforming the Olympics from an asymmetric financial risk for individual cities into a shared global investment where savings from eliminating redundant construction are redistributed to athlete development in under-resourced nations. The IOC currently returns approximately 90 percent of its revenue to NOCs and IFs worldwide, and this redistribution model is fundamental to the IOC's role in the global Olympic movement (19). Under the rotational model, billions of dollars that could support athlete development are instead lost to “white elephant” venues. For example, Beijing's Bird's Nest stadium costs $11 million annually to maintain despite limited use. Similarly, Rio's Olympic Park facilities are largely abandoned, and Athens' 2004 facilities sit underutilized despite their high quality. A permanent venue model would eliminate this waste and redirect those billions toward the IOC's global redistribution system, potentially doubling or tripling the support provided to NOCs in developing nations. This represents a shift from a model where wealthy cities compete for prestige while the Global South is structurally marginalized to a model where collective investment in shared infrastructure generates savings that enhance global equity.

Furthermore, a permanent Olympic site could function as a global high-performance hub accessible to athletes from all nations, particularly those from countries lacking advanced training facilities. Rather than sitting dormant between Games, the permanent site could operate year-round as an international training center where athletes from developing nations could access world-class facilities through Olympic scholarships and development programs. This addresses one of the profound inequities of the current model: Olympic training facilities are concentrated in wealthy nations, while athletes from developing nations lack access to comparable resources, creating systematic disadvantages that undermine the Olympic ideal of fair competition. A permanent site functioning as a shared global utility would provide athletes from all nations access to optimal training conditions, potentially enhancing competitive equity more effectively than the symbolic equity of rotating hosting opportunities among wealthy nations that cannot be accessed by the Global South.

The question becomes whether Olympic universalism is better served by a rotational model that in practice is mostly exclusive to wealthy nations and generates costs that could support athlete development, or by a permanent model that concentrates infrastructure in one location but significantly expands redistribution through mechanisms like Olympic Solidarity. We argue that the latter represents a more substantive form of equity than the symbolic equity of rotating hosting opportunities among wealthy nations, while systemic financial barriers effectively limit the Global South's access to both hosting opportunities and adequate development support. Beyond these material and economic equity concerns, a distinct critique addresses the symbolic and cultural dimensions of Olympic universalism.

### 5.2 Loss of global character and cultural representation

A second counter-argument concerns the potential loss of the Games' global character and reduced cultural representation if the Olympics are permanently located in Europe rather than rotating through different cultures and regions. This critique has merit—one of the appeals of the rotational model is the cultural diversity it brings, with each host showcasing its unique culture, architecture, and traditions, creating a sense that the Olympics belong to the world rather than to any particular place. Permanent hosting risks reducing this cultural diversity and creating a sense that the Olympics are a European rather than global institution, potentially alienating populations in other regions who no longer see the Olympics as relevant to their cultures and experiences.

However, this counter-argument must be weighed against several considerations. First, in an age of global media and transportation, physical location matters less for accessibility and global participation than it did in Coubertin's era—athletes and spectators from around the world can travel to a permanent site as easily as they can travel to rotating locations, and television and digital media provide global access regardless of physical location, meaning that permanent hosting would not significantly reduce global participation or viewership.

Second, the cultural showcase function of the Olympics could be maintained through other means even with permanent hosting—opening and closing ceremonies could feature different cultures on a rotating basis, cultural programs could highlight different regions, and the permanent site could incorporate architectural and artistic elements from diverse cultures rather than reflecting only European traditions. Such models already exist in other major international events: the Frankfurt Book Fair designates a “Guest of Honour” country each year, and the China International Industry Fair features a “Country of Honour,” allowing featured nations to showcase their culture, literature, innovation, and achievements without bearing the infrastructure burden of hosting the entire event. A permanent Olympic site could similarly adopt a “Nation of Honour” or “Continent of Honour” framework, rotating cultural representation through programming while maintaining infrastructural stability. The cultural benefits of rotation could be preserved through programming rather than requiring physical rotation of the entire event.

Third, the current rotational model's cultural benefits must be weighed against its costs. If the price of cultural diversity is systematic economic dysfunction, environmental degradation, and white elephant venues that burden host cities for decades, the question becomes whether these costs justify the cultural benefits, particularly when those benefits could be achieved through other means that do not require repeated construction in new locations. The cultural showcase of each Olympics lasts two weeks, but the debt burdens and abandoned infrastructure might last for decades—is this trade-off justified, or could cultural diversity be celebrated without incurring such costs?.

### 5.3 What about the Paralympic Games?

A related and highly legitimate concern involves the Paralympic Games. It is frequently argued—with considerable validity—that the rotational model serves as a powerful catalyst, compelling successive host nations to modernize their urban infrastructure to become more accessible and disability-friendly. Acknowledging the undeniable value of these localized accessibility legacies, a permanent site could alternatively establish an enduring global gold standard for universal design. Furthermore, the substantial capital saved from eliminating redundant construction could be systematically redirected to fund parasport programs and targeted accessibility initiatives worldwide, ensuring that disability inclusion is not exclusively tied to the wealth required to host a mega-event (24). Permanent hosting could also facilitate a proposal that has long been debated within Olympic circles yet remains logistically prohibitive under the current rotational model: the full integration of Olympic and Paralympic Games into a single unified event with separate but synchronous competitions (25). The operational complexity of coordinating two simultaneous major sporting events—involving approximately 15,000 athletes across over 50 sporting disciplines, each with distinct scheduling, venue, volunteer, and media requirements—creates a near-impossible logistical challenge for sporadic hosts that lack institutional continuity (26). What remains mission impossible under rotation—where organizing committees dissolve within months and institutional knowledge evaporates—could become operationally feasible under permanent hosting, where continuous learning, accumulated expertise, and stable organizational structures would transform this Herculean challenge into a sustainable reality. Each successive Games cycle would allow organizers to refine logistical protocols, optimize overlapping venue schedules, and develop specialized workforce expertise in managing dual-event operations—capabilities that dissolve entirely when organizing committees disband and staff depart within months of each Games. A permanent host could maintain year-round relationships with international disability sport federations and Paralympic athletes, ensuring that integration planning becomes embedded in institutional culture and operational systems rather than treated as an improvised afterthought by rotating cities confronting this challenge for the first and only time.

### 5.4 Feasibility challenges: governance, financing, and political will

Having examined three fundamental equity critiques—economic, cultural, and disability inclusion—a final set of counter-arguments concerns the practical feasibility of implementing any permanent hosting model. Would permanent hosting require governance innovations that are too complex to implement? Would the financing model be viable? Would there be sufficient political will to undertake such a transformation? These are legitimate concerns that require detailed examination of implementation pathways rather than dismissal as mere technical details.

Governance of a permanent Olympic site would require new arrangements to ensure Olympic neutrality. These arrangements must protect host nations from exploitation, maintain IOC authority, and respect host state sovereignty. The Karamanlis Plan's proposal for extraterritorial status provides a valuable historical model. In 1980, Greece offered land near ancient Olympia to become a permanent, neutral Olympic site. This territory would hold extraterritorial status similar to the Vatican or UN headquarters. Under this plan, the IOC would have functional autonomy within the Olympic territory, while Greece would provide external security, transportation, and infrastructure (11). This model has clear precedents in international law. Examples include the UN headquarters in New York and the International Court of Justice in The Hague. These cases demonstrate that extraterritorial status for international organizations is feasible and well-established in practice. A bilateral or multilateral agreement between the host nation(s) and the IOC could establish an “Olympic territory” with special status. This designation would insulate the site from domestic politics while ensuring independent international governance. Ultimately, such a framework would effectively neutralize the geopolitical vulnerabilities that currently plague the rotational model. Most importantly, it would protect athletes from the boycotts and diplomatic disputes that inevitably arise when the Games are hosted by sovereign nations engaged in global conflicts.

Financing permanent hosting could involve several mechanisms that distribute costs equitably while ensuring sustainable operations. Olympic nations could contribute to the capital costs of permanent facilities through voluntary contributions calibrated to their economic capacity, transforming the Olympics from a burden concentrated on individual hosts into a distributed global investment. This approach parallels—though differs institutionally from—how international organizations pool resources for shared infrastructure. The IOC generates substantial revenue from broadcasting rights and global sponsorships (7, 19), and under permanent hosting a larger proportion of these revenues could be allocated to facility maintenance, operations, and athlete development programs, rather than being distributed to rotating host cities. Permanent facilities could potentially generate revenue through year-round use for elite training, international competitions, sports tourism, and commercial events, with this revenue helping to offset operating costs. Over a multi-decade period, permanent hosting would likely cost substantially less globally than continued rotation, eliminating the repeated construction of similar facilities in different locations and avoiding the infrastructure abandonment that characterizes many post-Olympic sites (1, 7), thereby creating fiscal space for enhanced athlete development support worldwide.

Political will is perhaps the most significant feasibility challenge, as the IOC has historically resisted permanent hosting due to concerns about losing the marketing revenue and geopolitical influence inherent in the quadrennial bidding process. However, several factors suggest that political will may be more achievable now than in previous eras. The IOC's own “change or be changed” framing acknowledges that external pressures may force transformation if proactive change is not undertaken (19). Declining interest from potential hosts has fundamentally altered power dynamics, forcing the IOC to move toward negotiated arrangements with willing hosts rather than competitive bidding (9, 23). The IOC 2019 policy reform expanded host eligibility from individual cities to multi-city regions and even multiple countries, representing one step toward addressing infrastructure sustainability (23). However, climate change creates constraints that cannot be managed away through incremental reforms. UN Agenda 2030 (27) creates external accountability for sustainability performance. These convergent pressures create conditions where transformative alternatives may be more politically feasible than in previous eras when the IOC could rely on a steady stream of eager bidders.

## 6 Discussion and conclusion

Having established the theoretical framework, empirical evidence, and historical context, it is clear that the systemic challenges facing the Olympic Games extend far beyond the construction imperative alone. The persistent economic cost overruns, environmental degradation, and recurring political fragilities—including boycotts and geopolitical vulnerabilities—are deeply interwoven symptoms of the rotational model itself. Addressing these multidimensional sustainability crises requires structural alternatives rather than incremental reforms. While concentrated permanent hosting—whether in single locations like Athens or regional clusters like the Alps—offers one primary approach, it is not the only viable structural alternative.

Several intermediate models could potentially reduce systemic infrastructure waste while preserving varying degrees of geographic diversity and rotational principles. For instance, a limited rotation model could restrict hosting to a select roster of established cities, rather than opening the bidding to the entire globe. Building on this concept, Baade and Matheson (1) propose designating exactly four Summer Olympic and three Winter Olympic venues worldwide to rotate staging duties. This specific framework ensures that Olympic sports facilities maintain a useful life well beyond a single three-week event. By relying on world-class facilities that require only periodic technological upgrades, this approach could substantially curtail infrastructural waste while still preserving geographic representation.

Expanding on this logic, regional cluster models—where multi-city or multi-country regions host the Games (28)—could operate at a continental scale. Under this approach, designated Olympic clusters across different global zones—such as European, Asian, Americas, Oceania, and collectively financed African clusters to address historical exclusion—would rotate hosting responsibilities on 12- to 16-year cycles. This predictable rotation would allow the IOC to preserve cultural representation while securely concentrating infrastructural demands within proven hubs, provided that clusters in developing regions are supported by the global revenue-sharing mechanisms discussed earlier. Even within a rotating model, simulation studies demonstrate that sustainable hosting is achievable provided that hosts strictly utilize existing and temporary infrastructure. For example, an analysis of three diverse potential hosts—a Central European international region, Florida (USA), and Shanghai (China)—shows that all can host the Summer Olympics in a highly sustainable manner by maximizing existing infrastructure. This pragmatic strategy substantially reduces environmental and social impacts while potentially achieving financial profitability (23).

Rather than viewing this solely as a theoretical proposition, we can observe this shift occurring out of necessity in other mega-sporting events. Facing severe infrastructural and financial crises, both the Copa América and the Commonwealth Games are increasingly abandoning traditional open-rotation policies. Instead, they are pivoting to proven hubs with demonstrated capacity as a matter of institutional survival. Furthermore, hybrid approaches could acknowledge the Winter Games' vulnerability to changing climate realities, including higher winter temperatures and unsuitable precipitation levels (16). This could be achieved by transitioning them to permanent or semi-permanent locations, while allowing the Summer Games to continue under modified rotational frameworks. This staggered approach might make structural transformation more politically feasible by preserving traditional elements where they remain temporarily viable. However, all these alternatives raise significant equity concerns. These include concentrated hosting privileges among wealthy cities, the need for complex cross-border coordination, and perceptions of unequal treatment between the Summer and Winter Games. Consequently, careful attention is required to ensure benefits and burdens are distributed equitably.

Implementing any alternative model necessitates new governance arrangements, innovative financing mechanisms, and robust political coalitions. As Morris et al. (9) emphasize, mega-events must be anchored within a generation-long strategic plan spanning two to three decades to genuinely serve sustainable urban development. This plan must be supported by stable governance structures that outlive transient Organizing Committees. Empirical evidence from London 2012 and Rio 2016 confirms this governance challenge. While organizing committees excel at event delivery, their rapid dissolution—with 90% of staff departing within one month and 99% within four months (9)—prevents the translation of Olympic energy into long-term urban benefits. Even within a single host city, outcomes diverge dramatically based on governance continuity. For example, London's Queen Elizabeth Olympic Park has achieved what planners rarely accomplish: a thriving new urban district with multiple uses and a large park that people actually visit—success that remains evident more than a decade later (9). In contrast, London's youth sport inspiration goal failed dramatically: national obesity levels continued to rise, childhood obesity increased, and by 2015 sport participation had dipped below pre-Games levels, showing little evidence of marked progress (9).

Thiswithin-host variation demonstrates that legacy success depends heavily on governance continuity and institutional stability, rather than on host city characteristics. This level of stability is precisely what temporary organizing committees cannot provide, but permanent hosting structures could ensure. The Karamanlis Plan's historical proposal for extraterritorial status provides one template. This involves establishing “Olympic territories” with a special legal status analogous to UN headquarters. Such an arrangement would insulate sites from domestic political pressures while granting the IOC functional autonomy. Financing represents an equally complex challenge. Current models place primary costs on individual host cities, thereby perpetuating the “winner's curse” dynamic. Transformative models could distribute costs through assessed contributions from all National Olympic Committees, scaled proportionally to their economic capacity. Other options include allocating larger shares of the IOC's broadcasting revenues to facility construction or establishing long-term Olympic infrastructure funds.

Ultimately, the primary barrier to these structural alternatives is political, as the IOC has historically resisted permanent hosting proposals. However, the accumulation of systematic evidence regarding sustainability failures, rigid climate constraints, binding global accountability frameworks, and declining host interest have fundamentally altered the political calculus. These converging factors have pushed the IOC to acknowledge that transformation may be necessary (“change or be changed”). Senior Olympic stakeholders confirm this shift reflects existential pressure. As one IOC leader acknowledged, “they were going to get nobody bidding at some point.” This reality is forcing a fundamental reconsideration of the franchise model toward “cocreation” with hosts and dispersed regional hosting (9).

However, critical assessments suggest that even these latest reforms remain fundamentally inadequate. VanWynsberghe et al. (29) characterize the IOC's sustainability initiatives as symbolic gestures that function primarily as rhetorical cover for an institution in crisis, rather than as genuine operational transformations. Most significantly, the IOC has consistently declined to establish the accountability mechanisms necessary to ensure sustainability objectives are met, with no evaluation processes to monitor whether reforms achieve their stated goals during planning and hosting phases (29). This gap between reform rhetoric and institutional accountability reinforces the argument that incremental policy adjustments cannot overcome the structural contradictions embedded in the rotational model itself.

Recent scholarship has identified an inherent structural tension between the requirements of staging mega-events and the pursuit of sustainability objectives, characterizing these goals as fundamentally incompatible within the current Olympic model (29). While this analysis focuses on critiquing existing reforms, we argue that permanent or semi-permanent hosting offers a structural solution to these contradictions. By eliminating the construction imperative inherent in rotation, establishing continuous institutional governance, and creating long-term accountability mechanisms, permanent hosting addresses the systemic incompatibility that incremental reforms cannot resolve. This approach transforms Olympic sustainability from an aspirational rhetoric into an operational requirement embedded in institutional structures.

The persistence of these challenges confirms that the Olympic Games confront a sustainability crisis that a half-century of incremental reforms has failed to resolve. By framing this crisis as a “wicked problem,” this conceptual review has demonstrated that the systemic failures of the Games are structural symptoms. These failures—manifesting as profound cost overruns, severe environmental degradation, and recurring political fragility—are inextricably linked to the rotational hosting model. As long as the rotational hosting model relies on redundant infrastructure development across varying geopolitical contexts, it will remain fundamentally at odds with modern sustainability mandates. As established, the Olympic movement has reached a third critical historical juncture. In previous eras, proposals for permanent hosting were thwarted by nationalist anxieties or the institutional desire to preserve bidding leverage. Unlike those times, today's crisis is uniquely defined by uncompromising physical and political boundaries. The structural architecture of the Games can no longer be treated as an internal, voluntary governance matter insulated from external global realities.

While this paper utilized Athens and an Alpine cluster as heuristic devices, the broader objective is to reconsider the entrenched institutional dogma that geographic rotation is the sole guarantor of Olympic universalism. Indeed, ending the systemic capital waste associated with chronically underutilized mega-venues could paradoxically enhance, rather than diminish, global equity. Billions of dollars are currently lost to redundant, rapidly decaying infrastructure. Redirecting these funds toward athlete development and shared, high-performance global hubs offers a far more substantive realization of the Olympic ideal. This approach is much more effective than the symbolic, yet ultimately exclusionary, rotation of host cities predominantly among wealthy nations. Such a profound structural shift would not be an abandonment of Olympic tradition, but rather a return to its origins. For over a millennium, the ancient Games thrived precisely because they were rooted in the continuity, tradition, and sacredness of Olympia. If the modern Olympic movement is to secure its legitimacy and viability in the twenty-first century, it must reconsider whether the virtue of geographic stability should be rediscovered. Ultimately, this conceptual review is an invitation to the academic community, sports management practitioners, and policymakers to “think out of the box” regarding the future of mega-events. This entrenched “business-as-usual” logic remains tethered to decisions made over a century ago under vastly different economic and environmental realities. By questioning it, we can begin to seriously evaluate whether embracing alternative structural configurations offers the most viable pathway for achieving genuine sustainability and safeguarding the Olympic ideal for generations to come.
