# Correction: Thinking out of the (Olympic) box: is it time to reconsider permanent hosting for sustainable Olympics? A conceptual review

**DOI:** 10.3389/fspor.2026.1876252

**Published:** 2026-05-28

**Authors:** Athanasios (Sakis) Pappous

**Affiliations:** Department of Quality of Life Studies, University of Bologna, Rimini, Italy

**Keywords:** institutional governance, International Olympic Committee (IOC), legacies, Olympic games, paralympic games, sport mega events, sustainability, wicked problems

Reference 7 was erroneously written as Zimbalist A, Solberg HA, Storm RK. The Olympic economy on the edge: can the Olympic games survive its current financial model? In: Hautbois C, Desbordes L, editors. *in Research Handbook on Major Sporting Events*. Cheltenham: Edward Elgar Publishing) (2024). p. 40–54. It should be Zimbalist A, Solberg HA, Storm RK. “The Olympic economy on the edge: can the Olympic Games survive its current financial model?” In: Solberg HA, Storm RK, Swart K, editors. *Research Handbook on Major Sporting Events*. Cheltenham: Edward Elgar Publishing (2024). p. 693–708. doi: 10.4337/9781800885653.00065

Reference 10 was erroneously written as Albanidis E. Modern Greece and the olympics: politics and ideology. *Int J Hist Sport.* (2007) 24:93–104. doi: 10.3727/152599525X1741828722317. It should be Albanidis E, Barney RK, Choutas N. An Olympian sanctuary: the quest to permanently host the Olympic Games in Greece. *J Olympic Hist.* (2007) 15(2):28–39.

Reference 13 was erroneously written as Byers T, Hayday E, Pappous A. A new conceptualization of mega sports event legacy delivery: wicked problems and critical realist solution. *Sport Manag Rev.* (2020) 23:171–82. doi: 10.1007/BF01405730. It should be Byers T, Hayday E, Pappous A. A new conceptualization of mega sports event legacy delivery: wicked problems and critical realist solution. *Sport Manag Rev.* (2020) 23:171–82. doi: 10.1016/j.smr.2019.04.001

Reference 16 was erroneously written as Dean JW. Fake snow, faking sustainability: host selection and the winter Olympics' growing reliance on artificial snow ahead of Milan-Cortina d'Ampezzo 2026. *J Olympic Studies*. (2025) 6:64–82. doi: 10.5406/26396025.6.1.04. It should be Dean JW. Fake snow, faking sustainability: host selection and the Winter Olympics’ growing reliance on artificial snow ahead of Milan-Cortina d'Ampezzo 2026. *J Olympic Studies*. (2025) 6(1):46–65. doi: 10.5406/26396025.6.1.04

Reference 19 was erroneously written as International Olympic Committee. *Olympic Agenda 2020+5: 15 Recommendations*. Lausanne: IOC (2021). doi: 10.1080/17516230903027872. It should be International Olympic Committee. *Olympic Agenda 2020+5: 15 Recommendations*. Lausanne: IOC (2021).

Reference 22 was erroneously written as Darcy S, Taylor T. Managing Olympic venues. In: Frawley S, Adair D, editors. *in Managing the Olympics*. Basingstoke: Palgrave Macmillan) (2013). p. 125–42. doi: 10. 1057/9780230389588_7. It should be Darcy S, Taylor T. “Managing Olympic venues”. In: Frawley S, Adair D, editors. *Managing the Olympics*. Basingstoke: Palgrave Macmillan (2013). p. 99–126. doi: 10.1057/9780230389588_7

Reference number 28 was erroneously written as Kaspar R. Hosting mega-events in the Gulf. In: Reiche D, Brannagan PM, editors. *Routledge Handbook of Sport in the Middle East*. London: Routledge (2022). p. 419–32. doi: 10.4324/9781003032915-30. It should be: Kaspar R. “Hosting mega-events in the Gulf”. In: Reiche D, Brannagan PM, editors. *Routledge Handbook of Sport in the Middle East*. London: Routledge (2022). p. 276–286. doi: 10.4324/9781003032915-30

Reference 29 was erroneously written as VanWynsberghe R., Derom I., Pentifallo Gadd C. (2021). Legacy and sustainability in the Olympic movement's new norm era: when reforms are not enough. *Int J Sport Policy and Politics* 13, 255–75. doi: 10.1080/19406940.2021. 1898442. It should be VanWynsberghe R, Derom I, Pentifallo Gadd C. Legacy and sustainability in the Olympic Movement's new norm era: when reforms are not enough. *Int J Sport Policy Politics* (2021) 13(3):443–460. doi: 10.1080/19406940.2021.1898442

In the abstract, the words “Final Revised Abstract” were erroneously inserted in the first sentence. This has been corrected to read:

“The modern Olympic Games confront a sustainability crisis that a half-century of incremental reforms has failed to resolve.”

The original version of this article has been updated.

